# Supplementary material for: Typology of Parent-to-Child Emotions: A Study of Japanese Parents of a Foetus up to a 12-Year-Old Child
Source: Healthcare (Basel). 2024 Apr 24;12(9):881. doi: 10.3390/healthcare12090881 (PMC11083413; doi:10.3390/healthcare12090881)
Supplement: Supplementary file 1 [file healthcare-12-00881-s001.zip › healthcare-2935024-supplementary.pdf]

healthcare

MDPI

Supplementary Table S1. Cross table for each segment with cluster, and chi-squared test for parents' gender among parents belonging same range of child's age.

	Cluster 1: Lack of Bonding Emotions n = 509 (11.1%)	Cluster 2: Bonding Disorder n = 1471 (32.0%)	Cluster 3: Ambivalent Bonding n = 1211 (26.3%)	Cluster 4: Positive Bonding n = 1409 (30.6%)	χ^2 (df)
Segment	n (%)	n (%)	n (%)	n (%)	
1: 1st trimester, father (n = 126 [100.0%])	18 (14.3)	72 (57.1)	18 (14.3)	18 (14.3)	4.57 (3)^{NS}
2: 1st trimester, mother (n = 48 [100.0%])	8 (16.7)	21 (43.7)	6 (12.5)	13 (27.1)	
3: 2nd trimester, father (n = 210 [100.0%])	17 (8.1)	126 (60.0)	29 (13.8)	38 (18.1)	79.82 (3)***
4: 2nd trimester, mother (n = 216 [100.0%])	44 (20.4)	39 (18.0)	52 (24.1)	81 (37.5)	
5: 3rd trimester, father	51 (20.4)	64 (25.6)	28 (11.2)	107 (42.8)	13.42 (3)*
6: 3rd trimester, mother	49 (19.6)	34 (13.6)	42 (16.8)	125 (50.0)	
7: 0 to 1 months old, father	36 (14.4)	68 (27.2)	47 (18.8)	99 (39.6)	15.88 (3)**
8: 0 to 1 months old, mother	25 (10.0)	51 (20.4)	85 (34.0)	89 (35.6)	
9: 2 to 6 months old father	19 (7.6)	67 (26.8)	62 (24.8)	102 (40.8)	9.96 (3)*
10: 2 to 6 months old mother	18 (7.2)	42 (16.8)	87 (34.8)	103 (41.2)	
11: 7 to 17-months old, father	32 (12.8)	56 (22.4)	63 (25.2)	99 (39.6)	12.31 (3)**
12: 7 to 17 months old, mother	16 (6.4)	66 (26.4)	88 (35.2)	80 (32.0)	
13: 18 months to 2 years old, father	26 (10.4)	91 (36.4)	66 (26.4)	67 (26.8)	9.51 (3)*
14: 18 months to 2 years old, mother	10 (4.0)	94 (37.6)	84 (33.6)	62 (24.8)	
15: 3 to 5 years old, father	19 (7.6)	92 (36.8)	70 (28.0)	69 (27.6)	4.17 (3)^{NS}
16: 3 to 5 years old, mother	19 (7.6)	90 (36.0)	88 (35.2)	53 (21.2)	
17: 6 to 8 years old, father	29 (11.6)	86 (34.4)	66 (26.4)	69 (27.6)	15.17 (3)**
18: 6 to 8 years old, mother	19 (7.6)	112 (44.8)	80 (32.0)	39 (15.6)	
19: 9 to 12 years old, father	32 (12.8)	93 (37.2)	66 (26.4)	59 (23.6)	10.03 (3)*
20: 9 to 12 years old, mother	22 (8.8)	107 (42.8)	84 (33.6)	37 (14.8)	

Note. The number of cases belonging to each segment 5 to 20 is 250. *p < .05; **, p < .01 ***; p < .001; ^{NS}, not significant.

Supplementary Table S2. Cross table for combinations of parents' gender and child's gender with clusters.

combination Parents' gender × Child's gender		Cluster 1: Lack of Bonding Emotions	Cluster 2: Bonding Disorder	Cluster 3: Ambivalent Bonding	Cluster 4: Positive Bonding	total
		509 (11.1%)	1471 (32.0%)	1211 (26.3%)	1409 (30.6)	4600 (100.0)
		n (%)	*n* (%)	*n* (%)	*n* (%)	*n* (%)
Combination 1	Father x boys	127 (10.9)	418 (36.0)	264 (22.7)	353 (30.4)	1162 (100.0)
Combination 2	Father x girls	136 (13.1)	329 (32.0)	228 (22.2)	336 (32.7)	1029 (100.0)
Combination 3	Father x unknown	16 (11.0)	68 (46.9)	23 (15.9)	38 (26.2)	145 (100.0)
Combination 4	Mother x boys	103 (9.4)	322 (29.4)	346 (31.6)	325 (29.6)	1096 (100.0)
Combination 5	Mother x girls	106 (10.0)	303 (28.7)	330 (31.2)	318 (30.1)	1057 (100.0)
Combination 6	Mother x unknown	21 (18.9)	31 (28.0)	20 (18.0)	39 (35.1)	111 (100.0)

Note. χ^2 (*df*) = 296.64 (15), $p < .001$.
